# SN-38 Sensitizes BRCA-Proficient Ovarian Cancers to PARP Inhibitors through Inhibiting Homologous Recombination Repair

**DOI:** 10.1155/2022/7243146

**Published:** 2022-10-11

**Authors:** Shengbin Lin, Jiaxin Tian, Qiang He, Minyi Yang, Zuyang Chen, Alexey A. Belogurov, Xiao Li, Fan Zhang, Yongzhu Liu, Guo Chen

**Affiliations:** ^1^Department of Medical Biochemistry and Molecular Biology, School of Medicine, Jinan University, Guangzhou 510632, China; ^2^Department of Gynecology, The Sixth Affiliated Hospital of Guangzhou Medical University, Qingyuan People's Hospital, Guangdong 511500, China; ^3^Shemyakin-Ovchinnikov Institute of Bioorganic Chemistry of the Russian Academy of Sciences, Miklukho-Maklaya 16/10, 117997 Moscow, Russia; ^4^Tianjin Key Laboratory of Human Development and Reproductive Regulation, Tianjin Central Hospital of Gynecology Obstetrics, Tianjin 300100, China; ^5^The 5th Affiliated Hospital, Guangzhou Medical University, Guangzhou 510700, China; ^6^School of Biopharmacy, China Pharmaceutical University, Nanjing 211198, China

## Abstract

As a multifunctional protein posttranslational modification enzyme in eukaryotic cells, Poly-ADP-ribose polymerase (PARP) acts as a DNA damage sensor, which helps to repair DNA damage through recruiting repair proteins to the DNA break sites. PARP inhibitors offer a significant clinical benefit for ovarian cancer with *BRCA1/2* mutations. However, the majority of ovarian cancer patients harbor wild-type (WT) *BRCA1/2* status, which narrows its clinical application. Here, we identified a small compound, SN-38, a CPT analog, which sensitizes *BRCA*-proficient ovarian cancer cells to PARP inhibitor treatment by inhibiting homologous recombination (HR) repair. SN-38 treatment greatly enhanced PARP inhibitor olaparib induced DNA double-strand breaks (DSBs) and DNA replication stress. Meanwhile, the combination of SN-38 and olaparib synergistically induced apoptosis in ovarian cancer. Furthermore, combination administration of SN-38 and olaparib induced synergistic antitumor efficacy in an ovarian cancer xenograft model *in vivo*. Therefore, our study provides a novel therapeutic strategy to optimize PARP inhibitor therapy for patients with *BRCA*-proficient ovarian cancer.

## 1. Introduction

As the genetic material for all the living cells, DNA is fragile and easily damaged by endogenous and exogenous sources including reactive oxygen species (ROS), environmental and dietary carcinogens, and radiation [1]. In response to various types of damage, cells activate complicated signal cascades, which help the cell to repair the damaged DNA before dividing [2]. Cell fate after DNA damage was determined by factors involved in DNA damage recognition, repair, and injury tolerance, as well as activation of apoptosis, necrosis, autophagy, and senescence [3]. And these pathways that determine cell fate are not independent of each other [4]. The signaling pathways that are associated with DNA damage and repair play key roles in the initiation and progression of cancer [5]. They are also important in determining the outcome of cancer treatment with genotoxic drugs. Developing drugs or therapies based on the molecular basis of these pathways is important to optimize cancer treatment [6]. Currently, a number of cancer therapeutics are designed to induce unrepairable DNA damage in cancer cells, such as tumor radiotherapy and chemotherapy [7].

As a multifunctional protein posttranslational modifying enzyme, PARP catalyzes poly-ADP-ribosylation on various substrate proteins, and it is a key protein in base excision repair (BER) [8]. When DNA damage occurs, PARP1 and its homolog PARP2, which are the first responders of DNA damage, recognize the damage site firstly, and then, they recruit other repair proteins to complete the damage repair process [9]. PARP inhibitor binds to PARP1/2 and inhibits their enzymatic activity, resulting in the accumulation of unrepairable single-strand breaks (SSB) and finally transformed into the double-strand breaks (DSBs), which highly reply on homologous recombination- (HR-) mediated pathway to repair. Thus, cells with HR repair deficiency are particularly susceptible to PARP inhibition. Taking advantage of this principle, PARP inhibitor is developed, and it is the first anticancer drug successfully approved for clinical use by using the concept of synthetic lethality [10, 11]. Therefore, HR repair capacity is the primary factor that determines the PARP inhibitor efficacy; if the HR pathway is also dysfunctional at this time, it will produce a synthetic lethal effect, to have a stronger killing effect on tumor cells [12].

Synthetic lethality is a process in which defects in two different genes or pathways jointly lead to cell death. PARP inhibitor is the first FDA-approved anticancer drug, which utilizes this concept and specifically kills cancer cells with impaired HR repair capacity [13]. However, in *BRCA1/2*-proficient ovarian cancers, PARP inhibitors' therapeutic effects are relatively low [14]. How to improve the therapeutic effects of PARP inhibitor in *BRCA1/2*-proficient ovarian cancers is still an urgent problem needed to be solved at this stage [15]. In this study, we identified a compound SN-38, an analog of the natural compound camptothecin (CPT), potently inhibited HR repair activity and sensitize ovarian cancer cells to PARP inhibitor treatment *in vitro* and *in vivo*. SN-38 (7-ethyl-10-hydroxycamptothecin), a TOP1 inhibitor, is an active metabolite of irinotecan, which is widely used in ovarian cancer treatment [16–18]. Therefore, our study provided a novel strategy and potential drug candidate to optimize future PARP inhibitor therapy in ovarian cancer patients.

## 2. Materials and Methods

### 2.1. Cell Culture

Tow *BRCA1/2*-proficient ovarian cancer cell lines including A2780 and OVCAR3 were purchased from American Type Culture Collection (ATCC). Cells were grown in RPMI 1640 medium (ATCC modification) (Gibco, Thermo Fisher, USA) supplemented with 10% fetal bovine serum (FBS) (Hyclone, Thermo Fisher, USA) and 1% penicillin/streptomycin (Corning, USA). Each cell line was passage every 3 6 days. All cells were maintained at 37°C in a 5% CO_2_ and 95% air atmosphere incubator.

### 2.2. Reagents

Anti-*β*-actin (sc-47778) antibody was purchased from Santa Cruz Biotechnology (Santa Cruz, CA). Anti-Ki67 (#9027) and anti-cleaved caspase 3 (#9579) antibodies were purchased from Cell Signaling Technology (Danvers, MA). Anti-*γ*H2AX (05-636) antibody was purchased from Millipore (Billerica, MA). Anti-pRPA2 S33 (A300-246A) and anti-RPA2 (A300-244A) antibodies were purchased from Bethyl Laboratories (Montgomery, TX). Olaparib and SN-38 were obtained from Selleckchem (Houston, TX). Cells were transfected with indicated plasmids using Lipofectamine™ 3000 transfection reagent (Thermo Fisher Scientific, USA) according to the manufacturer's instructions.

### 2.3. HR Repair Reporter Assays

We used U2OS-DRGFP cells that harbor a chromosome-integrated DR-GFP reporter to measure HR efficiency. U2OS-DR-GFP cells were equally planted into two 60 mm cell culture dishes as the control group and the experimental group. Cells were transfected with 3 *μ*g of I-SceI expression plasmid pCBA-Sce-I using Lipofectamine™ 3000 (Thermo Fisher Scientific). 24 hours after the transfection, cells were treated with 1 *μ*M of SN-38 or DMSO, and 24 hours after treatment, cells were collected and subjected to flow cytometry analysis to determine percentages of GFP-positive cells.

### 2.4. Immunofluorescence Analysis

A2780 cells grown in the chamber slider were firstly irradiated with 10 Gy of radiation and then treated with or without SN-38 (10 *μ*M) for 2 hours. After treatment, cells were washed with PBS and fixed with 4% paraformaldehyde for 10 min at room temperature. Cells were then permeabilized with 0.3% Triton-100 for 10 min on ice. After extensively washing with PBS, cells were incubated with primary antibodies including Rad51 (1: 200) and *γ*H2AX (1 : 500) overnight at 4°C. After washing, cells were incubated with Alexa Fluor secondary antibodies (1 : 1000) at room temperature for 1 hr. Then, image acquisition was performed after washing with PBS and mounting with DAPI.

### 2.5. Colony Formation

Equal numbers of cells were seeded onto six-well plates in triplicate, treated with different concentrations of various compounds, and incubated for 14–20 days. Then, colonies were fixed and stained with 0.5% crystal violet. The colonies were counted using ImageJ software (NIH) or manually. All cell survival assays were performed at least in triplicate.

### 2.6. CCK8 Assay

Cell viability assay was performed using A CCK8 Kit (Beyotime, China). 5 × 10^3^ of cells were suspended with fresh solution and then seeded into 96-well plates. 24 hrs later, olaparib and SN-38 were added into each well. 48 hrs later, a 10 *μ*L of CCK8 agent was added into each well. The plates were incubated at 37°C for 1.5 hours, and then, the absorbance values at OD 450 nm were measured using an ELISA plate reader (BioTek, Winooski, VT, USA).

### 2.7. Comet Assay

The comet assay was performed using an OxiSelect™ Comet Assay Kit (#ADI-900-166, ENZO Life Science) according to manufacturer's instructions. Briefly, cells were plated into a 6-well plate and treated with olaparib, SN-38, or their combination. 24 hrs after treatment, cells were collected, washed, and resuspended in ice-cold PBS (without Mg^2+^ and Ca^2+^) at a final concentration of 1 × 10^5^ cells/mL. Then, we mixed 10 *μ*L of cell sample with 100 *μ*L of OxiSelect™ comet agarose and immediately transferred 75 *μ*L onto OxiSelect™ comet slides. The sliders were then placed at 4°C for 30 minutes and immersed in prechilled lysis solution for 30 to 60 minutes. After tapping off the excess buffer, the sliders were immersed in freshly prepared alkaline solution (pH > 13) for 60 minutes at room temperature, in the dark. Then, the sliders were electrophoresed in TBE buffer for 30 minutes at room temperature at 15 V (1 V/cm) and 300 mA. After electrophoresis, we dipped slide in 70% ethanol for 5 minutes and air dry samples and stained with 100 *μ*L/well of 1× Vista Green DNA dye in the dark for 30 min at room temperature. Slides were viewed with a fluorescence microscope (Olympus).

### 2.8. Annexin V/Propidium Iodide Staining

Apoptosis was measured using annexin V/PI costaining as previously described [19]. Briefly, A2780 or OVCAR-3 cells were treated with olaparib, SN-38, or their combination for 48 hrs. After treatment, cells were collected by centrifuge at 1000 rpm for 5 min and washed with PBS. The pellet was then resuspended in a 100 *μ*L binding buffer. Then, annexin V-FITC reagent and PI solution were incubated with each sample for 15 min in the dark at room temperature. Cell samples were then analyzed by flow cytometry (FACScan, BD Biosciences). Each sample was collected as 30,000 events and analyzed by FlowJo software (FlowJov10).

### 2.9. Cancer Xenograft Study

Six-week-old female nude mice were purchased from GemPharmatech Co., Ltd. (Nanjing, China) and housed under pathogen-free conditions. All the animal experiments were approved by the Institutional Animal Care and Use Committee of Jinan University. 1 × 10^7^ of A2780 cells was subcutaneously implanted into mouse flanks. When tumor volume reached around 100 mm^3^, tumor-bearing mice were randomly divided into 4 groups and orally administrated with olaparib (100 mg/kg), SN-38 (10 mg/kg), or their combination according to previous studies [20, 21]. We monitored tumor growth and measured tumor volume with a caliper every 5 days, and tumor volumes were calculated as V = (L × W^2^)/2 (L, length; W, width).

### 2.10. Immunohistochemistry (IHC)

Tumor sections were first deparaffinized with 100% xylene, followed by rehydration using gradient ethanol (100%, 90%, 70%, 30%, and 0%). After inactivation of endogenous peroxidase by 3% hydrogen peroxide and heat-based retrieval antigen in citrate buffer, IHC staining was then performed using R.T.U. Vectastain Kit (Vector Laboratories) according to the manufacturer's instructions. Primary antibody dilutions were anti-Ki67 (1 : 500), anti-*γ*H2AX (1 : 200), and anti-cleaved caspase 3 (1 : 200). All positive cells in tumor tissues were scored at 400x magnification. Percentage of positive cells was determined from three separate fields in each of three independent tumor samples.

### 2.11. Hematoxylin-Eosin (HE) Staining

Tissue damages including necrosis, congestion, and vacuolar degeneration were evaluated by hematoxylin-eosin (HE) staining as previously described [22]. Briefly, sliders were immersed in Harris hematoxylin solution for 10 seconds and then immersed in the eosin staining solution for 10-30 seconds after three times washing with water. After thoroughly washing with water, sliders were dehydrated by ascending alcohol solutions (50%, 70%, 80%, 95%, and 100%) and mounted.

### 2.12. Statistics

Data shown were from one representative experiment of at least three independent experiments and are expressed as mean ± SD. The statistical significance of the difference between groups was analyzed with a two-sided Student's *t*-test.

## 3. Results

### 3.1. SN-38 Inhibits Homologous Recombination (HR) in Ovarian Cancer Cells

**Figure 1:**
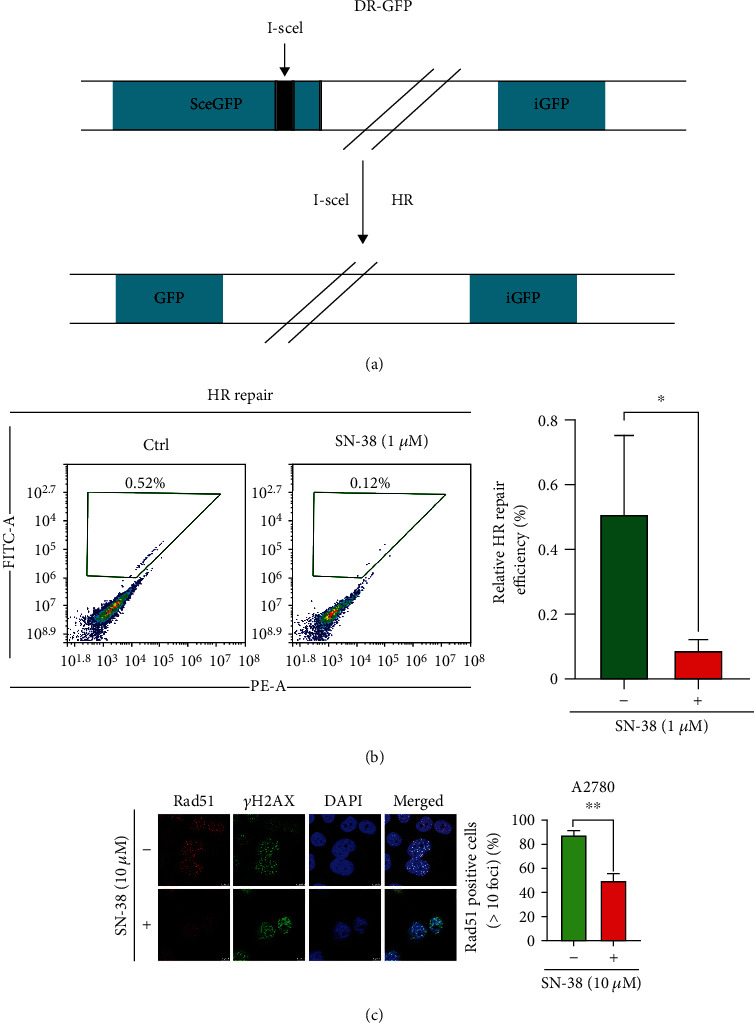
SN-38 inhibits homologous recombination repair in BRCA-proficient ovarian cancer cells. (a) Schematic diagram of HR reporter system. The expression of wild-type GFP can be rescued only by HR repair, resulting in GFP fluorescence. (b) HR repair activities were measured in cells treated with SN-38 (1 *μ*M) or control (Ctrl). Data represent the mean ± SD, *n* = 3 per group. ^∗^*P* < 0.05, by 2-tailed *t*-test. (c) Immunostaining analysis of IR-induced Rad51 in SN-38 (10 *μ*M) treated or untreated A2780 cells. ^∗∗^*P* < 0.01, by 2-tailed *t*-test.

The base excision repair (BER) is the primary pathway responsible for repairing single-strand breaks [23]. PARP1 is an important BER protein, and PARP inhibitor could disrupt BER by binding to the NAD^+^ catalytic site of PARP1 and subsequently caused DNA DSBs, which highly depend on HR pathway to repair [24]. If HR is inhibited at the same time, synthetic lethal effects could be produced [25]. Thus, HR activity could determine the PARP inhibitor sensitivity in cancer cells. We utilized the HR repair reporter system, which harbors an engineered GFP gene inactivated by insertion of the I-SceI endonuclease recognition site [26]. Only after the I-SceI-induced DSB is repaired by HR repair pathway, active GFP can be restored (Figure 1(a)). Thus, we can measure the HR repair activity by measuring the GFP expression. By using this system, we found that small molecule SN-38 significantly decreased levels of HR activity (Figure 1(b)). Rad51 recombinase catalyzes homologous pairing and strand exchange during HR and Rad51 foci are considered as the marker for HR repair [27]. To confirm that SN-38 could inhibit HR, we next evaluated the percentage of Rad51 foci-positive cells after SN-38 treatment by immunofluorescence assay. Our results showed that the percentage of Rad51 foci positive cells was significantly reduced in A2780 cells after SN-38 treatment (Figure 1(c)), which further validated that SN-38 inhibits HR.

### 3.2. Combination of SN-38 and Olaparib Synergistically Inhibits Ovarian Cancer Growth

**Figure 2:**
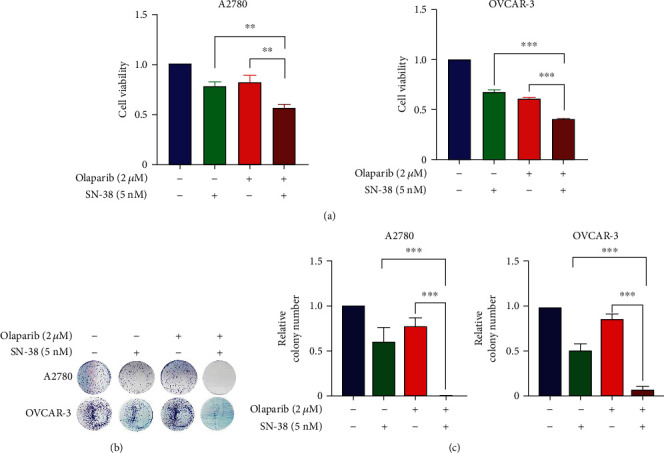
SN-38 sensitizes BRCA-proficient ovarian cancers to PARP inhibitors. (a) A2780 and OVCAR-3 cells were treated with 2 *μ*M olaparib, 5 nM SN-38, or their combination for 72 hrs, and the cell viability was measured by CCK8. (b, c) Colony formation survival analysis of A2780 and OVCAR-3 cells were treated with the indicated concentration of olaparib and SN-38. Representative colony formations were shown (b) and the relative number of colonies was quantified and normalized to untreated parental cells (c). ^∗∗^*P* < 0.01 and ^∗∗∗^*P* < 0.001, by two-tailed *t*-test.

Given that HR repair activity dictates olaparib sensitivity, we next evaluated ovarian cancer cell growth in presence of olaparib, SN-38 alone, or their combination. As shown in Figure 2(a), combination treatment of SN-38 and olaparib inhibited cancer cell growth greater than SN-38 or olaparib treatment alone. Meanwhile, the number of colonies formed by the combined treatment was also significantly reduced compared with that of the single treatment (Figures 2(b) and 2(c)). Thus, these results demonstrated that the antiproliferative effect of SN-38 and olaparib combination is a general phenomenon in *BRAC*-proficient ovarian cancer cells.

### 3.3. Combination of Olaparib and SN-38 Induced Greater DNA Damage

**Figure 3:**
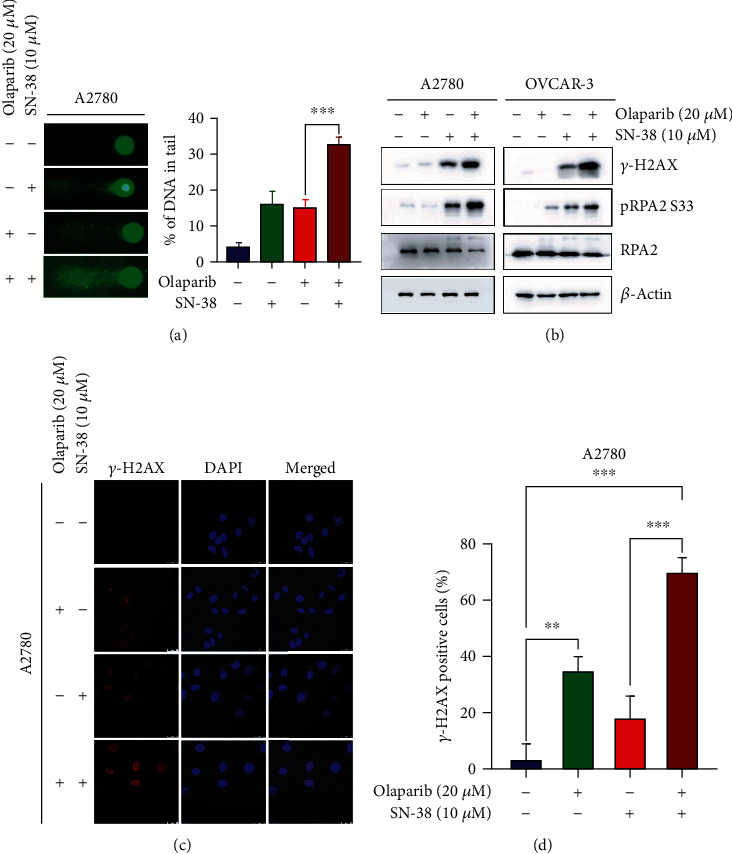
SN-38 and PARP inhibitor olaparib synergistically induces DNA damage and DNA replication stress. (a) A2780 cells were treated with the indicated concentration of olaparib and SN-38 for 24 hrs, followed by a comet assay of DNA damage. Representative image (left) and quantification of tail moments (right) were represented. (b) OVCAR-3 cells and A2780 cells were treated with indicated compounds, followed by western blot (b) and immunostaining (c, d) analysis of *γ*H2AX. Scale bar is 25 *μ*M. The percentage of *γ*-H2AX-positive cells (≥5 foci) and the number of *γ*-H2AX foci per cell was determined by counting at least 100 cells from each sample. Data were represented as the mean ± SD, *n* = 3 per group. ^∗∗^*P* < 0.01 and ^∗∗∗^*P* < 0.001, by 2-tailed *t*-test.

DNA damage plays an important role in cancer radio-chemotherapy efficacy, especially in PARP inhibitor efficacy. Excessive damages that exceed the DNA repair capacity of cells can lead to cell death [28]. Here, we determined whether the compound combination enhanced DNA damage using an alkaline comet assay for detection of both SSBs (single-strand breaks) and DSBs. As shown in Figure 3(a), compared to each single drug treatment, the combination of the SN-38 and olaparib generated markedly increased tail intensity in A2780 cells, suggesting that more severe DNA damage was induced in combination treatment.

*γ*H2AX is the phosphorylation of H2AX at its S139 site, which is considered as a sensitive molecular marker for DNA double-strand breaks (DSBs) [29]. We then measured *γ*H2AX levels after compound treatments by western blot and immunofluorescence assay. As shown in Figures 3(b)–3(d), we detected a greater level of *γ*H2AX in cells treated with two-drug combinations compared with SN-38 or olaparib alone. PARP inhibitor induced DNA DSBs primarily resulted from unrepaired single-strand breaks (SSBs), which are generated from accumulated DNA replication stress. Consistently, we also detected a significant increase in RPA2 S33 phosphorylation, which is phosphorylated by ATR when exposure of single-stand DNA and is extensively used as a surrogate marker for DNA replication stress [30, 31].

### 3.4. Combination of Olaparib and SN-38 Synergistically Induced Apoptosis

**Figure 4:**
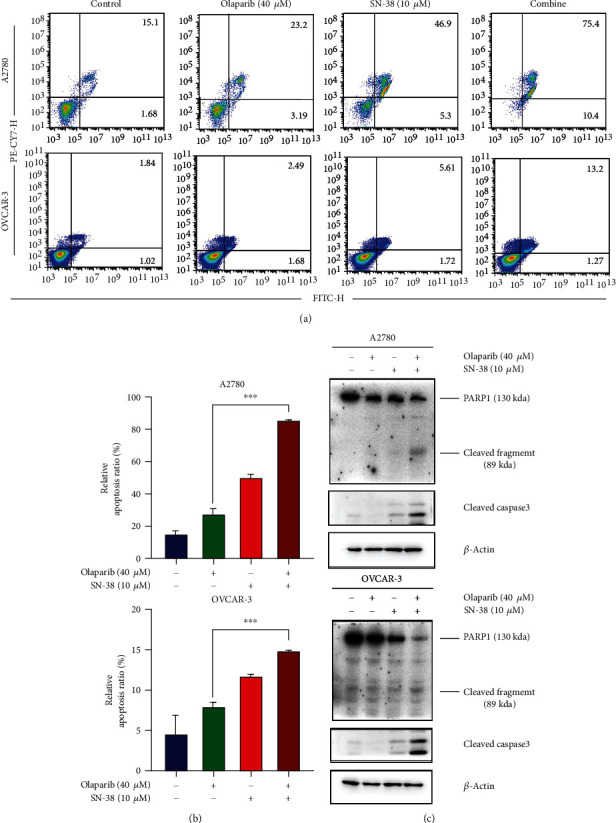
SN-38 and olaparib combination cause BRCA-proficient ovarian cancer cells apoptosis. A2780 cells were treated with 40 *μ*M olaparib, 10 *μ*M SN-38, or their combination as indicated, and cell apoptosis was analyzed at 48 hrs after treatment by annexin V staining (a, b) and western blot (c) analysis of cleaved caspase 3. Data were represented as the mean ± SD, *n* = 3 per group. ^∗∗∗^*P* < 0.001, by 2-tailed *t*-test.

DNA damage can lead to cell apoptosis whose activation is a key mechanism by which cytotoxic drugs kill tumor cells [32]. We conducted annexin V-PI staining and performed flow cytometry analysis to measure the cell apoptosis induced by drug treatments. As shown in Figures 4(a) and 4(b), the combined treatment led to a significant increase of the apoptotic population in A2780 and OVCAR3 cells compared to each compound treatment alone. Caspase 3 is a critical executioner of apoptosis, and it is cleaved into an active form during cell apoptosis [33]. As is shown in Figure 4(c), the combined treatment showed greater cleavage of caspase 3 and PARP1 than either SN-38 or olaparib treatment alone. These results demonstrated that the combination of SN-38 and olaparib induced extensive apoptosis in ovarian cancer cells.

### 3.5. SN-38 Enhances the Antitumor Efficacy of Olaparib in A2780 Xenografts

**Figure 5:**
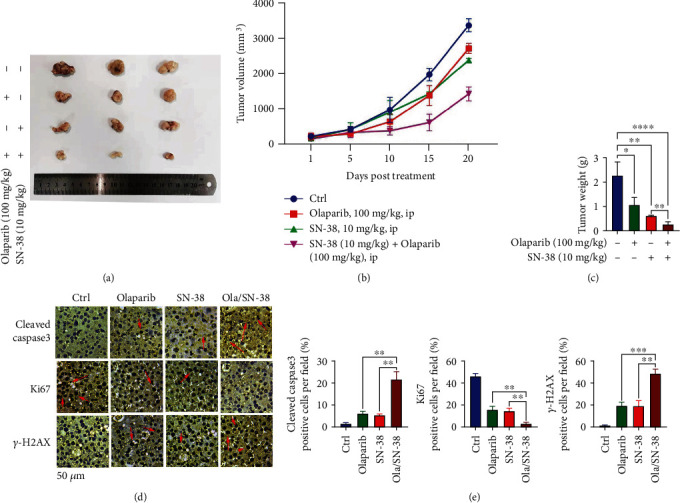
SN-38 enhances the antitumor efficacy of olaparib in an A2780 subcutaneous xenograft model. Mice bearing A2780 xenograft were divided into 4 groups, and each group received 100 mg/kg olaparib, 10 mg/kg SN-38 alone, or their combination; tumor volumes were measured every 5 days. Tumor volumes were measured (a) and tumor growth curve (b) was shown. Tumors were weighed and shown in (c). (d, e) Immunohistochemistry (IHC) analysis of Ki67 in the tumors derived from the 4 groups of mice mentioned above. ^∗^*P* < 0.05, ^∗∗^*P* < 0.01, and ^∗∗∗^*P* < 0.001 by 2-tailed *t*-test.

We then used A2780 ovarian cancer xenograft model to subsequently investigate the antitumor efficacy of the compound combination. SN-38 (10 mg/kg), olaparib (100 mg/kg), and their combination were administered to mice bearing tumors as described in Materials and Methods. Tumor volumes and body weights were measured every 5 days. As shown in Figures 5(a)–5(c), the use of SN-38 or olaparib alone resulted in a certain inhibition of tumor growth, while stronger antitumor efficacy was observed in the combination treatment. In addition, immunohistochemistry (IHC) analysis of the cell proliferation marker Ki67, apoptosis marker cleaved caspase 3, and DNA damage marker *γ*H2AX was performed to further evaluate the therapeutic efficacy of treatments. Inconsistent with tumor growth, Ki67 positive cells were dramatically reduced, while cleaved caspase 3 and *γ*H2AX-positive cells were increased, in tumor tissues from mice receiving combination treatment (Figures 5(d) and 5(e)).

### 3.6. Combination of SN-38 and Olaparib Exhibited No Obvious Toxicity

**Figure 6:**
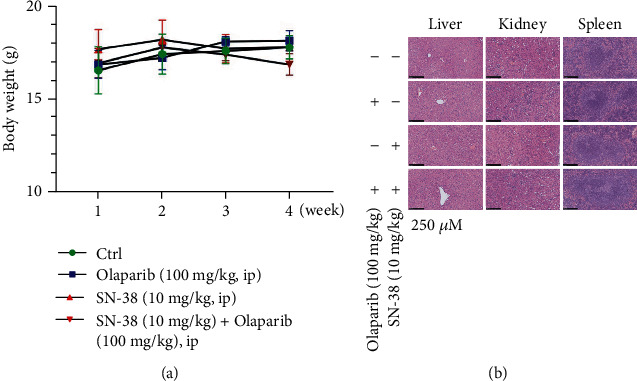
SN-38 and olaparib combination exhibits no obvious toxicity in mice. (a) The body weights of mice in each group were weighed every week, and the weight change curve was made. (b) HE staining histological analysis of paraffin-embedded sections of the liver, spleen, and kidney in each group.

We next evaluated the toxicity of treatments. Both SN-38 and the combination treatment did not cause a significant reduction in body weights (Figure 6(a)). Meanwhile, we also did not detect significant tissue toxicity on the liver, kidney, and spleen from mice treated with SN-38 alone or in combination with olaparib (Figure 6(b)). These results indicate that combination with SN-38 is a safe therapeutic strategy for PARP inhibitor therapy.

## 4. Discussion

PARP inhibitor is the first FDA-approved anticancer agent which utilizes synthetic lethality concept, and homologous recombination (HR) repair capacity is considered as the primary factor determining PARP inhibitor sensitivity. Developing agents inhibit HR repair which could render drug susceptible to PARP inhibitor insensitive cancer. Based on this premise, our studies demonstrated the first evidence that a combination of the PARP inhibitors and a small compound named SN-38, which individually have poor therapeutic effects, exhibited a greatly synergistic impact on *BRCA1/2*-proficient ovarian cancer. Since *BRAC1/2* genes play important roles in homologous recombination- (HR-) mediated DNA repair, thus, *BRAC1/2* mutant cancers are hypersensitive to PARP inhibitors. Mutations of *BRAC1/2* lead to the inhibition of cancer cell's HR repair capacity and the formation of synthetic lethal effects with PARP inhibitors. However, a significant number of cancers have normal *BRAC1/2* gene status, resulting in limited therapeutic efficacy for PARP inhibitors. Therefore, it is urgent to seek novel strategies to optimize PARP inhibitor therapy, such as in combination with other agents for *BRCA1/2*-proficient ovarian cancer. Here, we identified a small molecule SN-38, which could inhibit HR repair activity in ovarian cancer cells and verified the synergistic antitumor effects of SN-38 and olaparib combination in *BRCA1/2*-proficient ovarian cancer cells. Our data also showed that SN-38 combination with PARP inhibitors leads to significant accumulation of DNA damage as well as cell apoptosis, promoting cancer cell death. SN-38 exerts high potency against a variety of human cancers including ovarian cancer; however, its side effects and narrow therapeutic window hindered its monotherapy application in clinical therapy [34]. To exploit the therapeutic potential of SN-38, a number of antibody drug conjugate (ADC) preparations have been developed to ameliorate its adverse effects [35–37]. There are also some reviews of bioanalytical methods for SN-38 and some analyses from a clinical pharmacology perspective [38]. And the antibody-SN-38 conjugates are currently evaluated in phase II clinical trial on ovarian cancer patients [39]. Here, we show that SN-38 could be used as PARP inhibitor sensitizer and provide a novel strategy to apply SN-38 in future ovarian cancer treatment.

As a critical component of HR repair machinery, RAD51 facilitates DNA strand exchange and recombination. Our study suggests that the HR inhibiting activity of SN-38 was resulted or partially resulted from Rad51 recruitment. In addition, our results also showed that the combination of SN-38 and PARP inhibitor olaparib significantly caused replication stress, as well as apoptosis, in ovarian cancer cells. Thus, our findings suggest that a combination of PARP inhibitor with SN-38 could cause extensive DNA damage and DNA replication stress, subsequently leading to cancer cell apoptosis, therefore sensitizing *BRCA1/2*-proficient ovarian cancer cells to PARP inhibitors.

Taking together, our results herein demonstrated the synergistic effects of the PARP inhibitors and the SN-38 compound in HR-proficient ovarian cancer cells *in vitro* and xenograft tumors derived from *BRCA1/2*-proficient ovarian cancer cells in vivo, which do not respond well to the PARP inhibitors alone. Further, our findings provide evidence for the clinical development of PARP inhibitors in BRAC-proficient ovarian cancer patients.

## 5. Conclusions

Here, we identified a small compound SN-38, a CPT analog, which sensitizes BRCA-proficient ovarian cancer cells to PARP inhibitor treatment by inhibiting homologous recombination (HR) repair. In other words, our study provides a novel therapeutic strategy to optimize PARP inhibitor therapy for patients with BRCA-proficient ovarian cancers.

## Data Availability

The datasets used and/or analyzed during the current study are available from the corresponding author on reasonable request.
